# Incidental Detection of a Posterior Inferior Cerebellar Artery (PICA)-Terminating Vertebral Artery Variant During CT Evaluation of Venous Sinus Thrombosis: A Case Report

**DOI:** 10.7759/cureus.108584

**Published:** 2026-05-10

**Authors:** Shiraz I Syed, Mahesh Kumar, Raima Kaleemi, Imran Syed

**Affiliations:** 1 Radiology, Sofia Medical University, Sofia, BGR; 2 Radiology, Neurospinal and Cancer Care Postgraduate Institute, Karachi, PAK; 3 Radiology, Jinnah Postgraduate Medical Centre, Karachi, PAK; 4 Interventional Radiology, Basildon & Thurrock University Hospital, Mid and South Essex NHS Foundation Trust, Basildon, GBR

**Keywords:** hypoplastic vertebral artery, neuroradiology, posterior inferior cerebellar artery termination, vascular anomaly, vertebral artery (va)

## Abstract

A 25-year-old man presented to the emergency department with a sudden alteration in consciousness, characterised by reduced responsiveness to verbal stimuli and impaired neurological status. Baseline laboratory investigations demonstrated no evidence of coagulopathy, with coagulation parameters, prothrombin time (PT), international normalized ratio (INR), activated partial thromboplastin time (aPTT), and platelet count within normal reference ranges. Non-contrast CT of the brain revealed a left temporoparietal intraparenchymal haemorrhage with a left transverse sinus thrombus.

Arterial-phase CT angiography demonstrated a hypoplastic left vertebral artery (defined as a luminal diameter ≤2 mm or marked asymmetry relative to the contralateral vertebral artery) terminating as the ipsilateral posterior inferior cerebellar artery (PICA) without contribution to the basilar trunk, consistent with a PICA-terminating vertebral artery variant (PICA-VA).

The right vertebral artery was dominant based on a larger calibre relative to the contralateral side and continued as the basilar artery. No posterior-circulation aneurysm or arteriovenous malformation was identified.

In normal anatomy, the vertebral arteries fuse at the pontomedullary junction to form the basilar artery, with the PICA arising proximally. In this rare variant, one vertebral artery ends directly as the PICA rather than contributing to the basilar formation, an anatomic configuration that can mimic vertebral occlusion on imaging. Recognition of this pattern is important because it alters posterior circulation haemodynamics and may influence procedural planning or aneurysm risk assessment. In this patient, the PICA-terminating vertebral artery was an incidental finding during evaluation for venous sinus thrombosis. The variant did not alter management but clarified the posterior circulation anatomy and prevented misinterpretation as vertebral occlusion.

## Introduction

The vertebral arteries (VAs) unite to form the basilar artery at the pontomedullary junction, with the posterior inferior cerebellar artery (PICA) arising just proximal to this confluence, approximately V4 level [1]. In rare anatomical variants, a vertebral artery terminates directly as the PICA without confluence with the contralateral vessel, a configuration termed PICA-VA [1,2]. Once thought to be a rare anatomical anomaly (0.1-0.2%) [1], recent non-invasive imaging studies suggest a prevalence of 2.8-7% in general populations [2,3] and up to 18.7% in stroke cohorts [4]. This increased prevalence in stroke populations may reflect altered posterior circulation haemodynamics, as PICA-terminating vertebral arteries are associated with reduced vessel diameter, lower flow velocity, and increased vascular resistance [4]. Understanding and recognising this variation is essential, as it alters haemodynamic balance and has direct implications for stroke risk, aneurysm formation and endovascular planning [4-6].

## Case presentation

**Figure 1:**
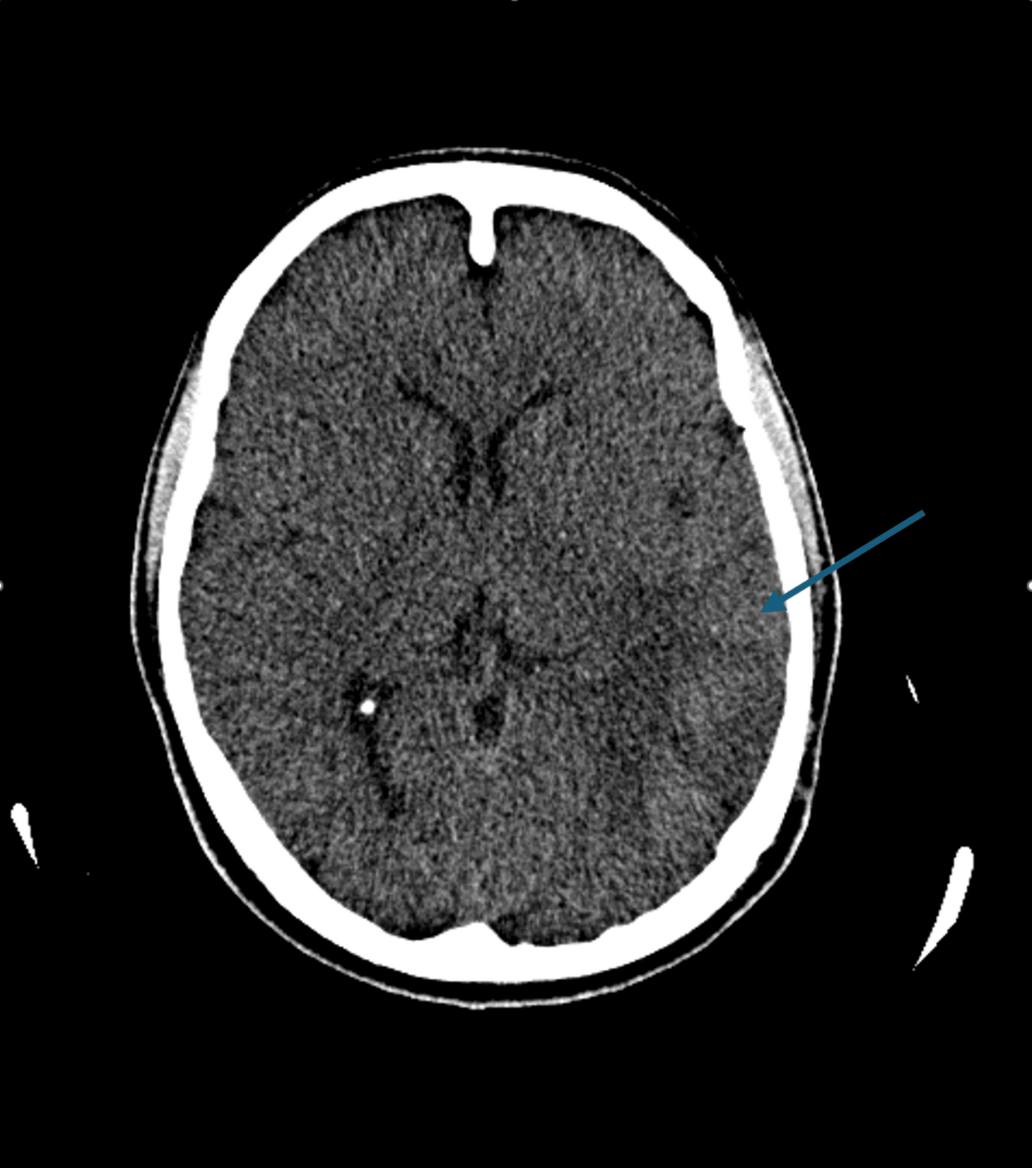
CT brain plain axial image showing left parietotemporal hemorrhage (blue arrow).

We describe a patient who presented with altered levels of consciousness secondary to a venous intracranial haemorrhage related to a left transverse sinus thrombosis. A non-contrast CT brain (Figure 1) demonstrated a left parietotemporal haemorrhage.

**Figure 2:**
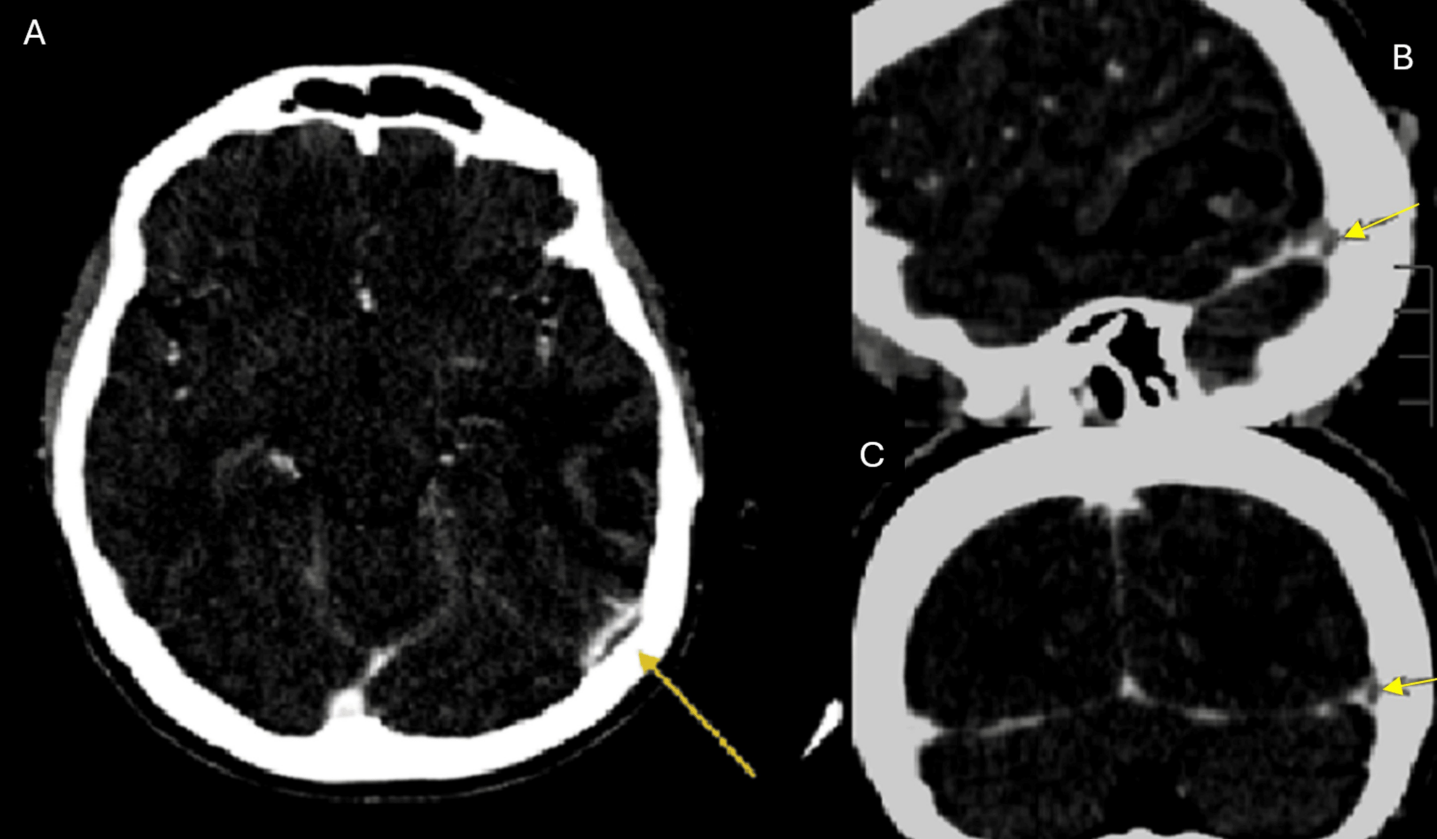
CT venography phase showing (A) axial, (B) sagittal, and (C) coronal views demonstrating a linear flow void in the left transverse sinus (Yellow Arrows).

**Figure 3:**
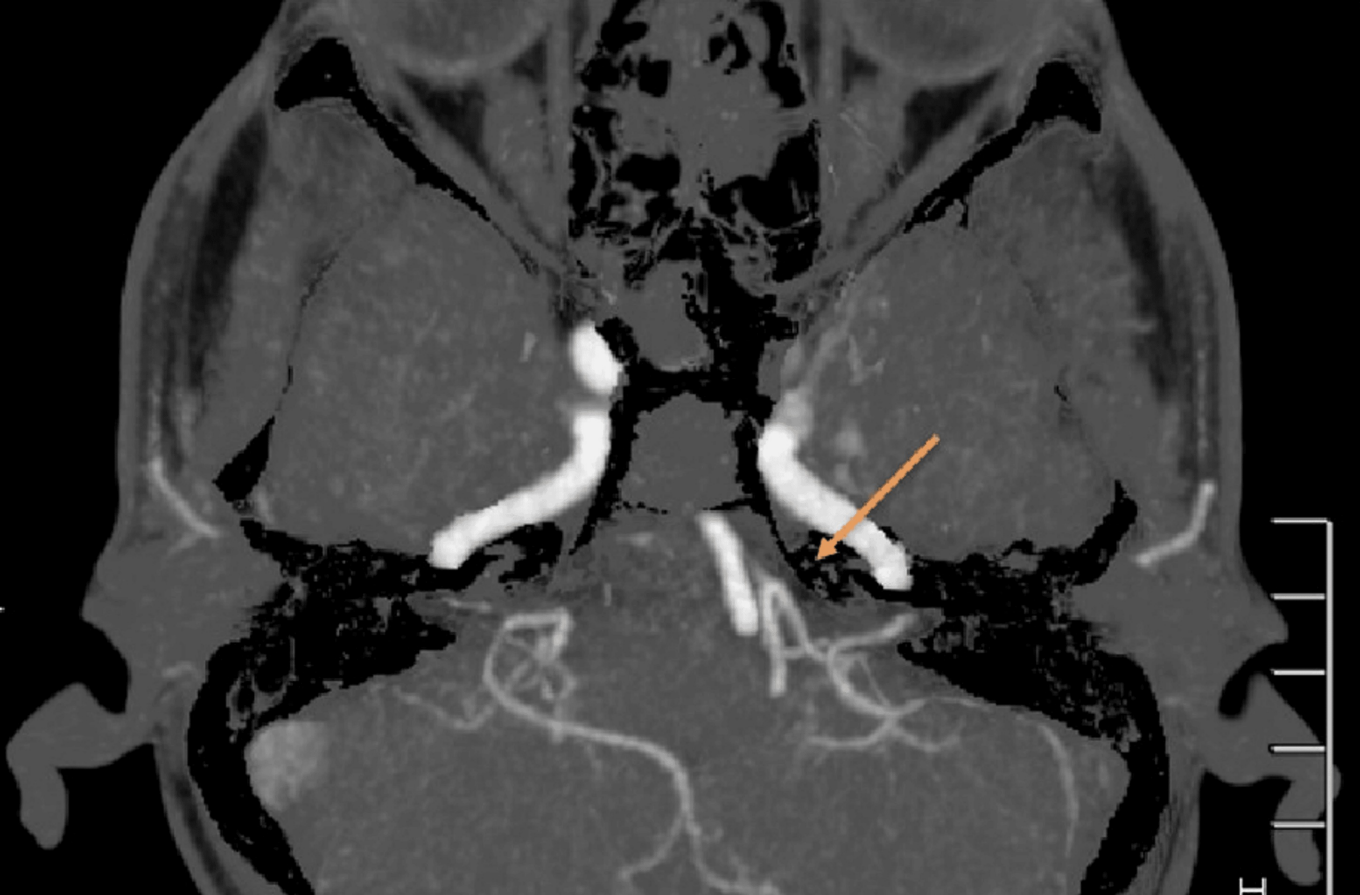
Maximum Intensity Projection axial section showing left vertebral artery terminating as PICA (orange arrow). PICA: Posterior inferior cerebellar artery

**Figure 4:**
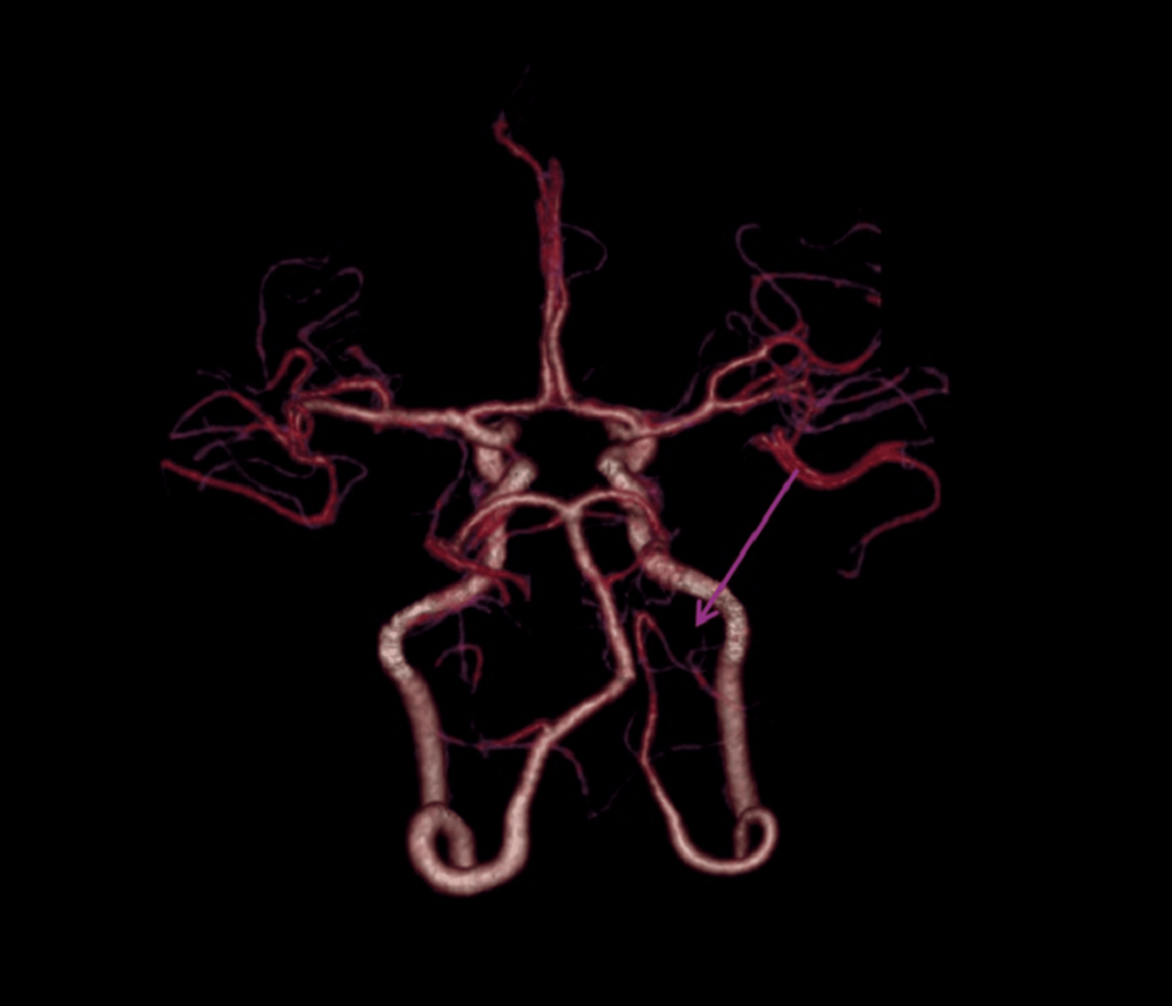
3D MIP image of Circle of Willis showing left PICA-VA (purple arrow). MIP: Maximum Intensity Projection

**Figure 5:**
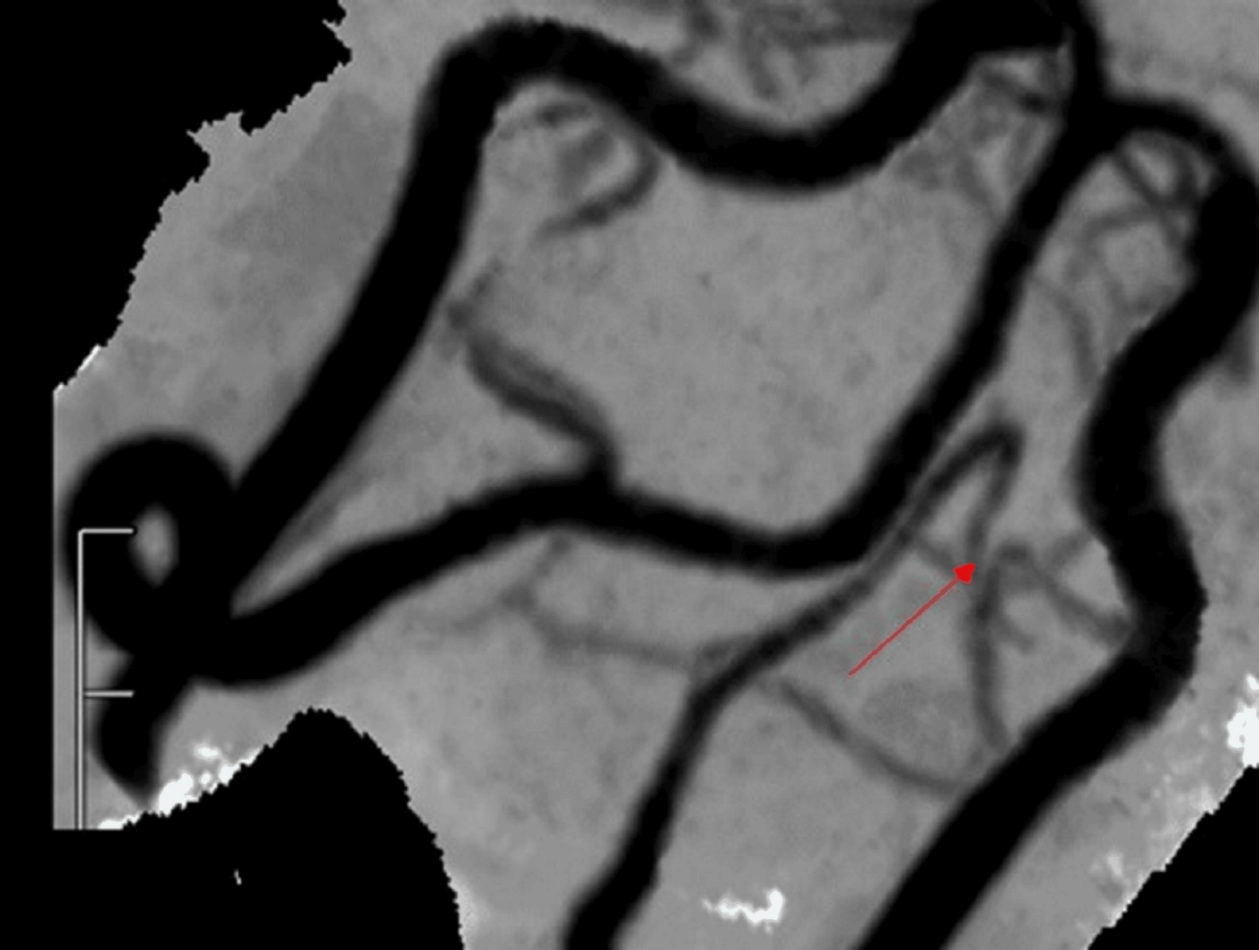
MIP angulated view showing left PICA-VA and a normal right vertebrobasilar system (red arrow). MIP: Maximum Intensity Projection

Subsequently, a comprehensive neurovascular evaluation was performed using CT angiography and CT venography (CTV) with Maximum Intensity Projection (MIP) reconstructions to assess vascular anatomy and venous outflow. The CTV phase showed a linear filling defect involving the left transverse sinus, which was hypoplastic, demonstrated by reduced calibre without intraluminal filling defect or abrupt cut-off, favouring a congenital variant rather than thrombosis (Figure 2). Additionally, these studies revealed a hypoplastic left vertebral artery terminating in the posterior inferior cerebellar artery (PICA), consistent with a PICA-VA variant (Figure 3), which clearly showed the left vertebral artery terminating as PICA. A 3D MIP reconstruction of the Circle of Willis (Figure 4) further delineated the variant, demonstrating the left PICA-VA configuration (purple arrow), while the contralateral vertebral artery continued normally to form the basilar artery. An additional MIP angulated view (Figure 5) illustrated the left PICA-VA variant in comparison with the normal right vertebrobasilar system, confirming asymmetry and hypoplasia of the left vertebral artery.

## Discussion

The termination of the vertebral artery (VA) into the posterior inferior cerebellar artery (PICA) is an unusual developmental abnormality. The PICA-terminating vertebral artery is thought to arise from persistence of embryonic vertebrobasilar configurations with incomplete distal fusion of the vertebral arteries, resulting in termination of one vertebral artery as the PICA rather than contributing to the basilar artery [4]. Usually, a dural ring allows the vertebral artery to enter the posterior fossa, where it joins the other vertebral artery to create the basilar artery. The cerebellar arteries are supplied by the vertebrobasilar system. The intradural, most distant branch of the V4 section of the vertebral artery is usually the posterior inferior cerebellar artery (PICA) [7]. The PICA can arise from the basilar artery, appear as a duplicated vessel, be absent in rare cases, or represent the terminal branch of the vertebral artery, as observed in our case [8].

The hemodynamics of the posterior circulation are changed by the PICA-VA termination variation. Patients may not experience any symptoms, but if collateral circulation is insufficient, the anomaly may put them at risk for pathophysiological consequences such as haemodynamic compromise, leading to ischemia [4], aneurysms [6] and Bow Hunter's Syndrome [9].

Crucially, if this variation is not thoroughly identified, it may mimic acquired VA blockage on angiography, resulting in a misdiagnosis. In this instance, the anomaly's angiographic identification guided appropriate care and avoided potential misdiagnosis [3].

Surgical methods for this region are viewed as hazardous because of the presence of numerous significant blood vessels and neural structures [10]. Strategies for intervention in PICA-VA termination necessitate careful preparation. Endovascular techniques like stenting or coiling pose a risk of unintended blockage of the PICA if it serves as the only endpoint of the VA [11].

Literature underscores that tailored approaches, guided by angiographic mapping, are crucial during vertebrobasilar endovascular procedures to avoid ischaemic complications [11].

Long-term results rely on identifying the anomaly and steering clear of procedures that jeopardize posterior circulation. Case studies and anatomical collections emphasize the infrequency of PICA-VA termination while underscoring its clinical significance. Our case adds to the limited body of evidence by demonstrating the incidental detection of a PICA-terminating vertebral artery in the setting of venous sinus thrombosis, where it may closely mimic vertebral artery occlusion on imaging and thus represents a clinically relevant diagnostic pitfall [4,11].

## Conclusions

Our case underscores how a seemingly rare vascular variant, PICA-VA, can surface unexpectedly in routine imaging. Although reported in up to 2.8-7% of healthy individuals, it is encountered rarely in clinical practice and may be mistaken for pathological occlusion. Recognition of this variant during venous sinus thrombosis work-up is essential to avoid misdiagnosis and reduce the risk of procedural complications, highlighting the need for vigilance in such cases.
